# Radical loss of an extreme extra-pair mating system

**DOI:** 10.1186/1472-6785-9-15

**Published:** 2009-05-19

**Authors:** Sjouke A Kingma, Michelle L Hall, Gernot Segelbacher, Anne Peters

**Affiliations:** 1 Behavioral Ecology of Sexual Signals Group, Max Planck Institute for Ornithology, Vogelwarte Radolfzell, Schlossallee 2, 78315, Radolfzell, Germany; 2 Department of Wildlife Ecology and Management, University of Freiburg, Tennenbacher Strasse 4, Freiburg, 79106, Germany

## Abstract

**Background:**

Mating outside the pair-bond is surprisingly common in socially monogamous birds, but rates of extra-pair paternity (EPP) vary widely between species. Although differences in life-history and contemporary ecological factors may explain some interspecific variation, evolutionary forces driving extra-pair (EP) mating remain largely obscure. Also, since there is a large phylogenetic component to the frequency of EPP, evolutionary inertia may contribute substantially to observed EP mating patterns. However, the relative importance of plasticity and phylogenetic constraints on the incidence of EP mating remains largely unknown.

**Results:**

We here demonstrate very low levels of EPP (4.4% of offspring) in the purple-crowned fairy-wren *Malurus coronatus*, a member of the genus with the highest known levels of EPP in birds. In addition, we show absence of the suite of distinctive behavioral and morphological adaptations associated with EP mating that characterize other fairy-wrens. Phylogenetic parsimony implies that these characteristics were lost in one speciation event. Nonetheless, many life-history and breeding parameters that are hypothesized to drive interspecific variation in EPP are not different in the purple-crowned fairy-wren compared to its promiscuous congeners.

**Conclusion:**

Such radical loss of an extreme EP mating system with all associated adaptations from a lineage of biologically very similar species indicates that evolutionary inertia does not necessarily constrain interspecific variation in EPP. Moreover, if apparently minor interspecific differences regularly cause large differences in EPP, this may be one reason why the evolution of EP mating is still poorly understood.

## Background

Social monogamy is the most common mating system in birds [1]. However, application of molecular tools revealed that most socially monogamous species engage in extra-pair (EP) mating (> 75% of studied species [2,3]). Rates of extra-pair paternity (EPP) are highly variable between species, ranging from none to almost all broods containing EP offspring [2-5]. The evolution of EP mating remains puzzling, largely because the generally proposed potential costs (reduced male care) and benefits (increased genetic quality of offspring) remain controversial [2,6].

When explaining variation among closely related species and between populations of the same species, EP mating rates are hypothesized to be a result of evolutionarily plastic responses to contemporary ecological factors. For example, lower breeding synchrony could reduce alternative mating opportunities and depleted genetic variation could diminish the genetic benefits of pursuing EP mating [2]. However, phylogenetic constraints on EP mating are important, with over 50% of the interspecific variation being explained at the level of families or orders [2,5], suggesting that contemporary ecological factors may (sometimes) play a secondary role [5]. Indeed, results of comparative studies may vary depending on whether phylogeny is taken into account (e.g., [7] vs. [8]). Nonetheless, the relative importance of phylogenetic constraints on one hand and plasticity on the other in determining interspecific variation in EPP rates remains largely unknown.

Here we examine the evolutionary flexibility of EPP rates by studying a member of a genus of birds that display an unusual mating system with extensive behavioral and morphological adaptations specialized for EP mating. Australian fairy-wrens (genus: *Malurus*) are considered the least faithful socially monogamous birds since all three members of the genus for which paternity has been analyzed show exceptionally high rates of EPP (see Table 1 and [9], with up to 95% of nests containing at least one EP offspring [10]. EP mating appears to be under female control, involving targeted pre-dawn forays to the territory of a preferred male, as shown in superb fairy-wrens (*M. cyaneus*) [11,12]. Male fairy-wrens invest heavily in mating competition as is evident from behavior as well as morphology. Before breeding, males molt into colorful breeding plumage [9] and develop unusually large testes and cloacal protuberances (the site of sperm storage), important for sperm competition [13]. Moreover, males engage in frequent courtship of EP females starting months prior to breeding and continuing all through the breeding season. EP courtship involves males intruding onto nearby territories followed by ritualized display of their bright breeding plumage during which a flower petal may be presented to the visited female. Territorial intrusions by extra-group males and petal displays have been described for eight of the nine Australian *Malurus *species [9], and therefore high EPP has generally been expected for all fairy-wrens (e.g., [9,13,14]).

**Table 1:** A comparison of rates of extra-pair paternity, and attributes of life-history, ecology and breeding biology in four *Malurus *species for which rates of extra-pair paternity are known.

	***M. coronatus***	***M. cyaneus***	***M. splendens***	***M. melanocephalus***
***Extra-pair paternity***				

% Broods containing EP offspring	5.8	83 – 95	55, 83	75, 63
% EP offspring	4.4	66 – 81	42, 73	56, 51
% Offspring sired by within-group subordinate	1.8	5	5, 8	*some*

***General biology***				

Number of helpers	1.3	0.9	0.6, 1.1	0.4
Number of male helpers	0.9	0.9	0.3, 0.6	0.4
Clutch size	3.0	3.2	2.9	2.8
Annual max # successful broods	2	3	2	> 1
% Annual pair-divorce	6.0	4.2	2.9	*low*
% Annual adult survival (male, female)	81, 81	75, 53; 67, 70	70, 59	
% Incestuous pairings	16.4	15	21.3	
Provisioning rate of males without helpers (feeds/h)	6.2	5.8, 6.4		
Months breeding	All, 1–2 peak(s)	Aug–Feb	Aug–Jan	Oct–Feb
Average breeding synchrony (max)	11.5 (28.2)	18.1 (29.8)		
Territory linearly arranged	Yes	No	No	No
Territory length in m	156	87	234, 237	262

In this study, we examine rate of EPP in purple-crowned fairy-wrens (*Malurus coronatus*). Purple-crowned fairy-wrens are riparian specialists rarely seen more than 20 m from the watercourse [9,15]. Year-round, groups vigorously defend a stretch of the stream that serves as their exclusive, all-purpose territory [16]. The cooperatively-breeding mating system of *M. coronatus *appears similar to other *Malurus *[9], but predictions of high rates of EPP for the species (e.g., [9,13,14]) may be premature. Although the species is less studied than most other fairy-wrens, extra-territorial display by males has never been observed and *M. coronatus *pairs coordinate song to form 'duets' [9,17], a feature generally related to low rates of EPP [18]. Here, we aim to establish the relative importance of phylogenetic constraints and evolutionary plasticity in response to ecological factors and life history, as determinants of extra-pair mating in *M. coronatus*. We quantify EPP rate and investigate male behavioral and morphological adaptations known to be important for EP advertisement and mating competition in other *Malurus *species. Additionally, we compare life-history and ecology of purple-crowned fairy-wrens with the other three species with known levels of EPP (superb, splendid *M. splendens*, and red-backed *M. melanocephalus *fairy-wrens). We consider attributes that have been hypothesized to affect costs and benefits of, and constraints on, EP mating (reviewed in [2-5]); in particular: social mating system [19] including number of helpers [10,14]; clutch size and nesting success [5]; divorce and mortality rates [5,20,21]; incidence of incestuous pairings [22,23]; importance of paternal care [5,24-27]; and breeding synchrony and density [7,28-30].

## Results

### Extra-pair paternity

In sharp contrast to the *Malurus *species studied so far (see Table 1), paternity analysis of 227 offspring from 104 nests in *M. coronatus *revealed very low levels of EPP: 4.4% of offspring (95% confidence interval CI: 1.7 – 7.1%) in 5.8% of broods (CI: 1.2 – 10.3%) resulted from extra-pair matings. These 10 EP offspring in 6 broods included 4 offspring sired by a subordinate male in 2 groups, thus corresponding to 6 extra-group offspring (2.6%, CI: 0.5 – 4.7%) in 4 broods (3.8%; CI: 0.1 – 7.6%). Paternity analysis was based on 6 microsatellite loci (2 to 17 alleles per locus) with a maternal exclusion probability of 94.2% and a paternal exclusion probability of 99.1% (Table 2). Microsatellite heterozygosity was high (range: 0.467–0.917; see Table 2).

**Table 2:** Number of alleles, observed (Ho) and expected (He) heterozygosity and exclusion probabilities of microsatellite loci used for paternity analyses in *Malurus coronatus*, based on 137 dominant birds.

Locus	No of alleles	Ho	He	Prob. of maternal exclusion	Prob. of paternal exclusion
Mcy1	6	0.796	0.734	0.326	0.502
Mcy3	9	0.810	0.830	0.494	0.666
Mcy4	2	0.496	0.500	0.123	0.187
Mcy8	17	0.917	0.912	0.688	0.816
MSp4	9	0.467	0.478	0.127	0.290
Msp6	7	0.684	0.704	0.292	0.469
Total	50			0.9423	0.9906

### Adaptations to extra-pair mating: morphology and behavior

**Figure 1:**
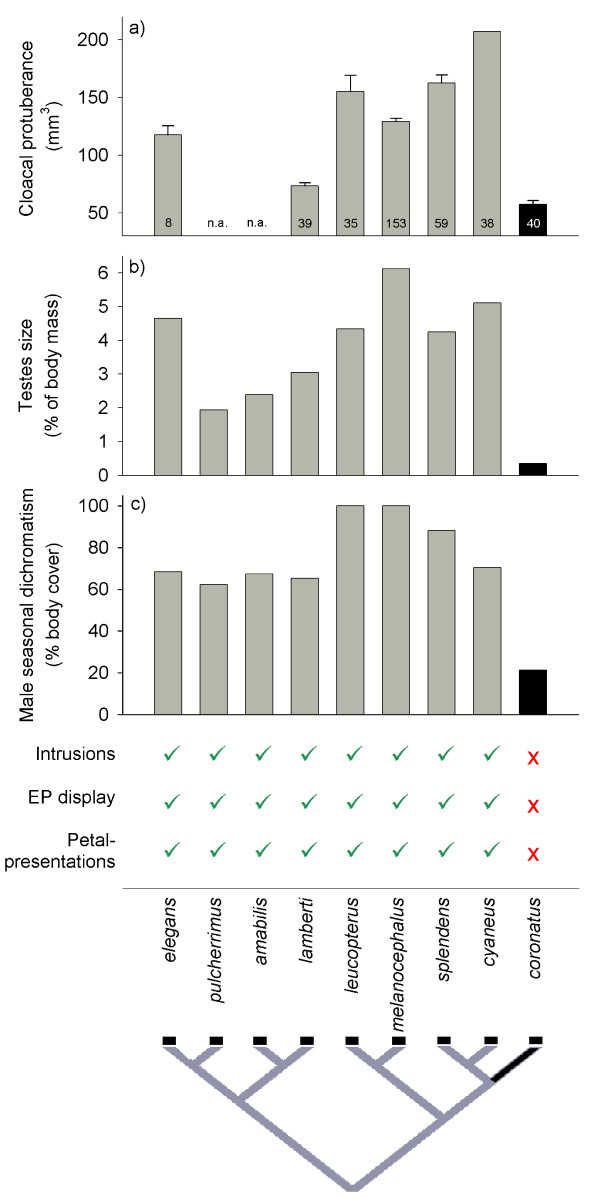
**Comparison of extra-pair mating adaptations in males of the nine Australian *Malurus *species**. For each species we illustrate (a) mean (± SE) size of the cloacal protuberance (CP) of breeding males (SE in *M. cyaneus *and data on CP size in *M. pulcherrimus *and *M. amabilis *were not available (n.a.); numbers depict sample sizes), (b) mean testes size (as % of body mass), (c) male breeding plumage cover (% of the body seasonally covered by colorful plumage), and (d) whether male extra-territorial forays, display behavior and petal presentations to extra-pair females, have been described (ticks) or not (crosses). Data on *M. coronatus *(dark bars) are from our study, except for testes size [61], and references for other data are given in the 'Methods' section. The phylogeny was obtained from [44].

In *M. coronatus*, we found none of the male morphological adaptations for EP mating that are characteristic of other members of the genus (Fig. 1). *M. coronatus *has much smaller reproductive organs during breeding (Fig. 1a, b): their testes are on average 10% of the size of testes of other *Malurus *(range: 6 – 18%; Fig. 1b) and cloacal protuberances (CP; the site of sperm storage) in breeding males (57.4 ± 3.6 mm^3^, n = 40) are on average about half of the CP size of other fairy-wrens (28 – 87%; Fig. 1a). A subset of males captured when their female was fertile (in the week before egg-laying) had an average CP of 66.3 ± 4.2 mm^3 ^(n = 25), a peak still much smaller than average breeding CP size in other *Malurus *(see Fig. 1a). Interestingly, *M. lamberti *and *M. pulcherrimus *appear to have rather small cloacal protuberances (see Fig. 1a, and [31]), although all other typical EP mating adaptations have been recorded (Fig. 1). It would therefore be interesting to explore EPP rates in these two species.

In addition, to the primary reproductive traits important for sperm competition, traits involved in behavioral sexual competition were also reduced in *M. coronatus*. The amount of body plumage which males molt seasonally into colored breeding plumage (21%) is only 21–34% of other *Malurus *(62–100%; Fig. 1c). Likewise, the striking male behavioral adaptations specific to EP mating in other fairy-wrens are absent: in over 325 hours of observations on fertile females, we never observed male *M. coronatus *engaging in extra-territorial intrusions, courtship displays or presentation of flower petals (Fig 1d). In contrast, previous research showed that fertile *M. cyaneus *females were visited by up to 7 intruding males per 20-min observation period, with more than 25% of females visited by at least one intruder [32].

### Comparing *Malurus *life-history and ecology

We compared *M. coronatus *with three congeners that have high EPP, concerning aspects of general breeding biology, life-history and ecology that have been proposed or shown to affect relative costs and benefits of and constraints on extra-pair mating (Table 1). This comparison showed that social mating system, number of helpers, clutch size, breeding success, rates of divorce, adult mortality, and incestuous pairing, rates of paternal care, and peak breeding synchrony are all very similar (Table 1).

All fairy-wrens are cooperative breeders, with subordinate birds assisting the dominant (breeding) pair with offspring provisioning (Table 1). Groups average 3.3 birds (range 2–11; n = 39) in *M. coronatus*, including male and female subordinate birds, with sex of subordinates being skewed towards males (around two third of subordinates were males). This results in approximately the same number of males present per territory compared to *M. cyaneus *(both 1.9 male per territory), and slightly more than in *M. splendens *(1.3–1.6) and *M. melanocephalus *(1.4; Table 1).

Clutch size was 3.0 ± 0.1 eggs per clutch on average (range: 1 – 4, n = 157 nests) intermediate to other fairy-wrens (range 2.8–3.2 eggs; Table 1). Like other fairy-wrens, *M. coronatus *can raise more than one successful brood annually (Table 1; [33,34]).

Pair bonds in all *Malurus *species are long-lasting for two reasons. First, divorce rates in *Malurus *are relatively low (Table 1). In our population, three of 25 surviving pairs from 1 April 2006 and none of 24 pairs from 1 April 2007 divorced (average annual divorce rate: 6.0%). This is similar to other species: in *M. cyaneus *4.2% of 93 pairs surviving to one year later had divorced [35], and in *M. splendens*, 10 pairs divorced in 348 pair-years (2.9%) [36]. Second, mortality rates in Malurus are generally low [9]. In our *M. coronatus *population on average 19% of dominant males and females did not survive until one year later (Hall et al., *in prep*) resulting in comparable to slightly higher survival rates than in other *Malurus *species.

We observed nine incestuous pairings of 55 pairings with known pedigree (16.4%; five mother-son, two father-daughter, and two full-sib pairings). This is similar to the potential for inbreeding in *M. cyaneus *and *M. splendens *(Table 1), where incestuous pairings occurred in 75 out of 500 pairings (15.0%; [37]) and 29 out of 136 pairings (21.3%; [38]) respectively.

Nestling feeding rates are similar in *M. coronatus *compared to *M. cyaneus*, the only congener with data available for comparison (Table 1). In *M. coronatus*, males in unassisted pairs have a provisioning rate of 6.2 feeds/hour (n = 25 males at 34 nests), which is intermediate between two measures from one population of *M. cyaneus *(5.8 and 6.4 feeds/hour [39,40].

The average breeding synchrony index of *M. coronatus *(based on 252 breeding attempts) was 36% lower than in *M. cyaneus *(Table 1), the only congener available for this comparison. This is because breeding in *M. coronatus *could occur in any month. However, there were typically peaks in spring (Aug – Sep) and in the wet-season (Dec – Apr), so peak synchrony was almost identical in both species (28.2 and 29.8% respectively).

Breeding density is determined by territory size and arrangement. Territory size in *M. coronatus *(average length = 156.5 ± 14.3 m, range: 50–376, N = 39 territories) is intermediate to other fairy-wrens (Table 1). However, in contrast to other fairy-wrens which have territories arranged in a mosaic (Table 1), territories in *M. coronatus *are linearly arranged along the stream and have therefore fewer neighbours.

## Discussion

EPP is a defining feature of the mating system of Australian fairy-wrens (genus *Malurus*), associated with striking behavioral and morphological adaptations. Although high infidelity has been assumed to occur in all fairy-wrens (e.g., [9,13,14]), we here show a marked exception: *M. coronatus' *mating system rather approaches genetic monogamy, with EPP rates being even lower than the average 18.7% of broods observed in socially monogamous birds [2]. Although levels of EPP in *Malurus *fluctuate between species as well as within species (e.g., in *M. splendens*: between populations 55 – 83% [14,22]); between years 33–63% [14] of nests), all studied species show high levels of promiscuity, with even the lowest values of EPP being far higher than that of *M. coronatus *(Table 1).

Contrary to all other fairy-wrens, *M. coronatus *males show reduced sexual signaling (reduced breeding plumage and the absence of courtship display), in line with lower levels of EPP [41]. Moreover, while other *Malurus *have some of the largest testes and cloacal protuberances known for birds [13], *M. coronatus *has relatively small reproductive organs, suggestive of reduced sperm-competition [42,43]. In fact, their testes are well below the average 1% of body mass for songbirds [42] and cloacal protuberances of breeding males are also smaller than expected for their body size [43].

A phylogeny including 9 of 12 species in the *Malurus *genus [44] suggests that *M. coronatus *is derived from an ancestor with high EPP and the adaptations to EP mating that characterize its sister species (Fig. 1). Thus, phylogenetic parsimony suggests that EP mating and the entire suite of adaptations were lost within a single speciation event (see also Box 1 in [45] for an example).

### Comparing *Malurus *life-history and ecology

Hypotheses attempting to explain interspecific variation in EPP usually consider differences in social mating system or contemporary ecology as important explanations that can affect potential benefits, costs and constraints of EP mating [2]. However, the radical loss of extreme levels of EPP in *M. coronatus *is not associated with any marked differences in relevant life-history parameters.

Clutch size could affect EPP rates [5], but average clutch size is very close to on average around 3 eggs in all species (Table 1), including *M. coronatus*. Similarly, differences in annual fecundity could affect rates of EPP [5], but all *Malurus *species can annually raise more than one successful brood (Table 1).

Potential (genetic) benefits of EPP and EPP rates can be dramatically affected by differences in mating system and opportunity to express social mate choice ([46,47]; see also [19]). However, all fairy-wrens are socially monogamous with restricted opportunities for social mate choice since breeding vacancies arise rarely in these long-lived, year-round territorial species with low divorce rates (see [9] and Table 1). Low rates of divorce and mortality in *M. coronatus *are associated with a low EPP rate, consistent with the pattern found in comparative studies [5,20,21]. While divorce rates are consistently low in all *Malurus *species, mortality rates are slightly lower in *M. coronatus *than in its sister species (Table 1). However, mortality rates are in general very low in the (promiscuous) *Malurus *species (see Figure Two in [5]), and, in fact, much lower than would be predicted from their high EPP rates [5]. Thus, it seems unlikely that a relatively slight decrease in adult mortality was important for the near-disappearance of EP mating in *M. coronatus*.

EP mating can act as a mechanism to reduce the costs of incest and inbreeding [23]. Philopatry increases the risk of incest and inbreeding in general, as it does in other fairy-wrens [22,23,33,37,48]. The frequency of incestuous pairings in purple-crowned fairy-wrens does not differ compared to the sister species (Table 1), including *M. splendens *for which inbreeding avoidance has been proposed as one of the benefits of EPP [22,23]. Therefore, in combination with the fact that molecular genetic variability is rather high (see Table 2), opportunities for genetic benefits of EP mating seem as likely in *M. coronatus *as in other fairy-wrens.

Some life-history attributes can constrain females in EP mating behavior. For example, the importance and extent of male care can affect rates of EPP [5,26]. All fairy-wrens are cooperative breeders with, on average 1–2 subordinates (Table 1) assisting the dominant (breeding) pair. In *M. cyaneus*, helpers liberate females from constraints on EP mating by compensating for the reduction in male care associated with EP behaviors [10]. Likewise, in *M. coronatus*, parents can decrease their feeding rate of nestlings when subordinates are present (unpubl. data). Increased value of male care in *M. coronatus *compared to other *Malurus *seems unlikely since nestling starvation is uncommon (unpubl. data), and nestling feeding rates of males without helpers are similar in *M. coronatus *compared to *M. cyaneus *(Table 1). In addition, male paternity-guarding behavior might limit EPP [49]. However, mate guarding is no more intense in *M. coronatus *than in *M. cyaneus *(see [17] for a detailed discussion), and in *M. cyaneus *male mate-guarding does not prevent EP mating by females [11,32].

Availability of suitable EP mating partners and hence EP mating opportunities might be reduced by asynchronous breeding [29] and low density [8]. Although *M. coronatus *breeds more irregularly and has a longer breeding season than other *Malurus *species, peak synchrony in *M. coronatus *is similar to peak synchrony in *M. cyaneus *(Table 1), its sister species that has the highest rates of EPP reported in birds [2]. Breeding density (territory size) at first appears similar. However, spatial arrangement of territories is different in purple-crowned fairy-wrens (see below).

It is difficult to demonstrate causation for a single evolutionary event, but a possibility is that the riparian specialization of *M. coronatus*, resulting in a one-dimensional arrangement of territories, may constrain access to an adequate sample of EP mates. Extra-pair offspring in *M. cyaneus *are generally sired by males within one or two territories (maximally five [11]). Territory length in *M. coronatus *is intermediate between the other fairy-wrens and the number of males per territory is similar (see Table 1). Therefore, if female purple-crowned fairy-wrens traveled similar distances in search of EP males, this linear arrangement implies that a female could choose from fewer potential sires. For example, for average group size and composition of *M. coronatus *and *M. cyaneus *(Table 1), within a two-territory radius there would be 7.6 males in 4 linearly arranged territories in *M. coronatus*, compared to 34.2 males in 18 territories in a honeycomb arrangement in *M. cyaneus *(see Figures in [12,15,34]). Male advertisement would be subject to analogous constraints since male *M. coronatus *would have fewer females to display to within a similar flight distance. However, inter-specific evidence for breeding density effects remains poor [2,5,8,50]. Moreover although extremely low breeding density will necessarily limit EP mating (cf. [51] and [52] for an example), it is not immediately obvious why a relatively reduced availability of EP mates should lead to virtual female fidelity and absence of male EP mating adaptations in *M. coronatus*. However, this hypothesis could be explored by examining whether differences in EP mate availability (population density) explain some of the variation in *Malurus' *rates of EPP between study years, populations and species.

## Conclusion

Although EP mating can have a strong phylogenetic component [2,5], our results show that remarkable differences in EP mating system and associated adaptations can occur among species with very similar life-histories. This pattern suggests that phylogenetic constraints do not necessarily limit rapid evolutionary modification of EPP rate, and that EP mating and its adaptations can be evolutionary much more labile than is commonly assumed [2,5]. If inter-specific variation in EP-levels is commonly not related to descent, social mating system, or obvious contemporary ecological factors, this could well be one reason why general explanations for the evolution of EPP have remained elusive [2-4].

## Methods

### Study species

We studied a color-banded population of *M. coronatus *resident along Annie Creek and the Adcock River in the Australian Wildlife Conservancy's Mornington Wildlife Sanctuary (S17° 31' E126° 6') in Western Australia. Like other fairy-wrens, *M. coronatus *are small (~10–12 g), sedentary, cooperatively breeding passerine birds. The dominant pair, the male and female that sing duets [9,16], form exclusive long-term pair bonds, and breed together. Subordinate birds are usually progeny from previous broods, and contribute to nestling feeding [9]. During regular weekly censuses in August to November 2005 and April 2006 to April 2008, we noted which group members were present and searched for nest-building females, identifying any intruders, and all interactions, including display behavior. Nests were checked during incubation to determine clutch size. At time of banding, a small blood sample was collected by brachial veni-puncture from nestlings (n = 164) and fledglings that were still dependent on their parents (n = 48), and stored in Queens- or Longmire's lysis buffer for paternity analyses (see below). In addition, material from unhatched eggs (n = 8) and dead nestlings (n = 7) was collected and stored in ethanol. We collected paternity data throughout the study period and area, covering the entire range of breeding synchrony and population density.

For the interspecific comparison with other *Malurus *(see below), we collected the following breeding parameters for *M. coronatus*, adhering to methods published for the other species (see also below). We measured cloacal protuberance (CP) height (h), width (w) and length (l; measured as the distance from the anterior to the posterior edge) to the nearest 0.1 mm of males captured in full breeding plumage and calculated volume following [43,53]. Average clutch size and annual number of broods raised to fledging was calculated over the period April 2006–April 2008. We calculated average number of helpers using data from 39 territories in October 2007. Divorce rates were calculated annually (from 1 April) as the percentage of pairs that had switched mates twelve months later, while both individuals survived (see also below). Actual annual divorce rates may be higher than estimated by this method because it does not correct for pair bond duration and is based on pairing at certain arbitrarily chosen times (for detailed discussion see [37]. However, this commonly used metric provides a comparable index of divorce rates. Annual adult mortality rates were calculated as the percentage of dominant birds that were not present in our study populations one year later. We can assume these individuals died, as surveys in adjacent areas revealed that (long-distance) dispersal by dominant birds is rare (Hall et al., unpubl. data).

To calculate % incestuous pairings (parent-offspring and full-sib pairings) we included all pairs for which we knew the relatedness of the dominant male and female (n = 55). Incestuous pairings do not necessarily result in inbreeding, because they frequently end in divorce (see Figure Four in [33] for *M. cyaneus*). Nevertheless, the occurrence of incestuous pairings does indicate potential for inbreeding which could be avoided by EP mating.

We collected nestling food provisioning rate of males without helpers (n = 34 nests of 25 males) by observing nests (with 1–4 nestlings between 4 and 10 days old) for 60 minutes and calculated feeding rates as number of provisioning trips per hour (Kingma et al., in prep).

Breeding synchrony was based on all broods over the study period and calculated using the formula [54,55]:



where SI_p _is the synchrony index for each female p (higher % means more females breeding in the population), *f*_i,p _the number of fertile females (excluding female p) in the population on day i, and t_p _the number of fertile days for female p, (defined as 6 days before the start of laying till the day of the penultimate egg), and F the total number of dominant females present during female p's fertile period. For every day when at least one fertile female was present, the average for every fertile female was calculated.

Purple-crowned fairy-wrens are riparian specialists with territories linearly arranged along the stream (see illustration in [15]), so territory size was approximated as the length of the stream occupied by the group determined by behavioral observation, based on GPS coordinates, in October 2007.

### Comparing *Malurus *species

#### Malurus phylogeny

We used a phylogeny based on allozyme data [44] showing three main clades (see Fig. 1), with *M. coronatus *as sister species to *M. splendens *and *M. cyaneus *all in one clade, confirmed by recent DNA analysis (J. Gardner & J. Trueman, pers. comm.). We included only the nine Australian *Malurus *species in this study, because there is very little information available about the three Papua New Guinean *Malurus *species (see [9]).

#### Extra-pair paternity

Published EPP data are available for *M. cyaneus, M. splendens*, and *M. melanocephalus *(see Table 1). For *M. splendens*, we present rates of EPP for two different populations [14,22], for *M. melanocephalus *we present two estimates from the same population [56,57], and for *M. cyaneus *we report the range of rates of EPP from one population collected over 15 years [10,11,37,58-60]. Since extra-group (rather than extra-pair) paternity was usually reported in *M. cyaneus*, we calculated rates of EPP as the percentage of offspring sired by a male outside the group, and added 4.9% within-group offspring sired by subordinate males, as reported in [60]. Since the percentage of broods in which subordinate males sired offspring has not been reported, the value presented is % broods with extra-group young, and may hence be a slight underestimation of the % broods with EP young. The percentage of offspring sired by within-group subordinates was not reported for *M. melanocephalus*, but like in other fairy-wrens, few offspring are sired by within-group subordinates (M.S. Webster and J. Karubian pers. comm.).

#### Reproductive organs and breeding plumage

For the comparison of relative testis size in all *Malurus *(including *M. coronatus*) we used the relevant specimen data used by [61]. Their standardized protocol for comparative analysis included at least five breeding males for each species, and used only individuals with enlarged testes for tropical species with variable breeding seasons (for details see [42,61]). We compiled data on CP size from original sources [53,57,62-64]. To make data on *M. coronatus *comparable with data on other species, we omitted inclusion of data on males that were not in breeding condition, as could be indicated by non-breeding plumage (e.g., in subsets in [57,62]). Similarly, we excluded a study in which CP length was measured to the cloacal vent instead of to the anterior edge [31], leading to smaller CP sizes.

Males in all fairy-wren species are seasonally dichromatic, alternating dull non-breeding plumage with bright-colored breeding plumage for several months of the year. Breeding plumage cover (% of the body with seasonally dimorphic plumage) of males of each species was estimated based on illustrations in [9] and [65]. Using The Gimp 2.2 , we manually selected the area covered by males' seasonal dimorphic plumage on the drawings and calculated the number of pixels (using a 'histogram'). A similar procedure was used to calculate number of pixels of the total bird and the percentage breeding plumage was calculated by: (number of pixels of seasonally dimorphic plumage/number of pixels total bird) * 100. Tail-feathers were not included. The average of three measures was used to determine mean percentage breeding plumage. The percentage presented in Fig. 1d, was obtained by averaging the two mean values based on both sources [9,65]. Repeatability for three measures of the remaining seven species was high [66] for the 3 repeated estimates within each source ([9]: repeatability = 99.5, F = 617.9, P < .001; [65]: repeatability = 99.9, F = 2555.2, P < .001) as well as for the 2 averages between sources (repeatability = 92.3, F = 25.0, P < .001); since breeding plumage covers 100% of the body in two species (*M. melanocephalus *and *M. leucopterus*), they were not included in the repeatability calculations.

#### Breeding biology

Data about general breeding biology, life-history and ecology of all *Malurus *species for which EPP data are available (Table 1) was, if possible, acquired from the same studies reporting EPP [10,11,14,22,37,56-60] and from a comprehensive review [9]. The following data were not available from these sources and were obtained from other studies, where possible from the same population in which EPP was studied: group- [67] and territory-size in *M. melanocephalus *and *M. splendens *[68,69], % inbreeding in *M. cyaneus *[37], number of annually raised broods [33,70] and annual divorce rates in *M. cyaneus *and *M. splendens *[35,36]. Divorce rates in *M. melanocephalus *are low, but not yet quantified (M.S. Webster, J. Karubian, pers. comm.). Comparable data on nestling feeding rate was only available for dominant *M. cyaneus *males without helpers [39,40]. We calculated breeding synchrony in *M. cyaneus *from original data on 87 females from the 1996 and 1997 breeding season (see [71]), following the same procedure as for *M. coronatus *(see above). Average territory length (diameter) in other fairy-wrens was calculated from area (in ha.) assuming a circular shape, to compare with territory size in *M. coronatus*.

### Paternity analyses in *M. coronatus*

#### DNA Extraction and Genotyping

Total genomic DNA was extracted from blood samples of dominant and subordinate birds, offspring, and tissue samples of eggs using standard salt-extraction described in [72]. The samples were genotyped by Ecogenics GmbH (Zurich, Switzerland) using a set of six microsatellite loci, which were previously used for paternity analyses in other fairy-wrens (Mcy μ1, Mcy μ3, Mcy μ4, Mcy μ8, developed for *M. cyaneus *[73], and Msp4, Msp6, developed for *M. splendens *[14]; see references for *genbank *numbers). Four microsatellite loci were included in a PCR multiplex (Mcy μ1, Mcy μ3, Mcy μ4, Msp6 with fluorescently labeled reverse primers). The other two loci (Mcy μ8 and Msp4) were used in single PCR reactions. PCR amplifications were optimized for a 10 μl reaction volume containing 2 μl of DNA, 5 μl master mix (Qiagen, Cat. No 206143 for multiplex and Cat. No 203445 for single PCR; containing Hotstar polymerase, PCR buffer, and dNTPs), 1.5 μl double distilled water, and 0.3 μM of forward and reverse primers each. The following thermo treatment was used on a TC-412 Programmable Thermal Controller (Techne): 35 cycles with 94°C for 30 seconds, 50°C for 90 seconds, and 72°C for 60 seconds. Before the first cycle, a prolonged denaturation step (95°C for 15 min) was included to activate the Hotstar enzyme, and the last cycle was followed by a 30 min extension at 60°C.

Genotyping was performed on an ABI PRISM 3100 Genetic Analyzer. The amplified PCR products (1.2 μl) were mixed with 10 μl formamide containing GENESCAN-500 (LIZ) Size Standard (Applied Biosystems), and the genotype was determined on an ABI PRISM^® ^3100 Genetic Analyzer using GeneScan Analysis^® ^Software 3.7 and Genotyper^® ^3.7 Software (Applied Biosystems).

All 346 individuals included in the paternity analysis had four (n = 3), five (n = 25) or all six (n = 318) loci typed.

#### Determination of parentage

In total, we genotyped 227 offspring from 104 broods. We used CERVUS v 3.0 software [74,75] to analyze paternity data. The expected (He) and observed (Ho) heterozygosity were calculated for each locus. We calculated heterozygosity (and parental exclusion probability, see below) using 137 dominant birds only, because genotypes of nestlings and subordinate birds (in most cases offspring from previous broods) were not independent.

None of the loci deviated from Hardy-Weinberg equilibrium and we did not find evidence for null alleles. Number of alleles ranged from 2 – 17 per locus and heterozygosity was high (Table 2). We calculated for each locus the probability of maternal and paternal exclusion, i.e., the probability of exclusion of a randomly chosen male or female as parent of the offspring, based on allele frequencies of dominant birds. In total across all 6 loci, we had 50 alleles and the probability of wrongly assigning a randomly chosen male as the sire was less than 1% (Table 2).

For paternity analyses, we assumed the social mother to be the genetic mother of the offspring. This assumption was justified since social mother and offspring had no mismatches except for one mismatch at a single locus, probably due to a scoring error (as suggested by homozygosity at the maternal locus). We assigned paternity using the following conservatively chosen parameters in CERVUS: number of candidate fathers = 20, proportion of males sampled = 0.9083 and frequency of typing error = 0.01. First, we tested whether the social (expected) father was likely the genetic father, by examining whether there were mismatches between the social father and offspring. In total 217 of the 227 offspring matched all paternal alleles, and were assigned as true sire with confidence of > 95% in all cases. Of the ten remaining offspring (from 6 nests, 4 nests with one, and 2 nests with three offspring not sired by the dominant male) with a paternal mismatch, one had a mismatch at one allele, six at 2 alleles, two at 3 alleles and one at 4 alleles. In the single case of one locus mismatch, it was likely that a subordinate male from the same group was the sire of the offspring, rather than a mutation or scoring error, because the subordinate male had no mismatches. Three other EP-offspring could be assigned to subordinate males from the same social group as the dominant male (in total one subordinate male sired one offspring, and one subordinate male sired three); both subordinates were unrelated to the dominant female. For the other six offspring not sired by their social father, we identified five dominant and one subordinate male from nearby groups as the genetic fathers (these matched the offspring's paternal alleles at all loci). Thus, in total, out of 227 offspring and 104 broods, we identified six extra-group offspring in four broods, and four instances of paternity by a subordinate male within the group in two broods, and thus ten EP offspring in six broods.

Paternity assignment may be particularly complex in cooperative species with high male philopatry, due to the presence of close relatives, which share some alleles with the expected father [72,73]. To verify our assignment of the social male as the sire of the 217 offspring that had no mismatches with their social father (thus excluding the 10 EP offspring), we used a likelihood-based approach. CERVUS calculates the likelihood for each male that he is the sire (LOD score) based on the offspring and maternal genotypes; the male with the highest LOD score is the most likely sire. We included all males (of at least 3 months old) within five territories as potential fathers and computed LOD scores for each candidate father. Using this method, we could confirm the social father to be the sire in 201 cases (he had the highest LOD score of all candidate males). For the remaining 16 cases, one (n = 12) or two (n = 4) other males showed higher LOD scores than the social father (despite no mismatches between offspring and social father). We estimated the probability of falsely accepting a social father as sire as *P*_*ep_offspring *_× *P*_*ep_mate *_= 0.27% (if one male had higher LOD) and 0.54% (if two males had higher LOD). *P*_*ep_offspring *_is the unequivocal probability that a given offspring is not sired by its social father = 4.7% (10 EP offspring and 201 within-pair offspring unambiguously assigned by allelic exclusion as well as LOD scores of social sires, see above). *P*_*ep_mate *_is the probability that the female would have mated EP with the one or two males in the population that had higher LOD than the social father by chance. We estimated *P*_*ep_mate *_by considering as potential sires all males older than 3 months within five territories distance from the focal territory, including the subordinate males within the focal territory, which were 17 alternative males in this subset. Thus, *P*_*ep_mate *_was on average 5.9% (for one male with higher LOD) and 11.8% (for two males with higher LOD) respectively.

## Authors' contributions

SAK: collected field data, carried out paternity and comparative analysis, and wrote the paper; MLH: collected field data, supervised field research, and contributed to writing; GS: supervised paternity analysis, and contributed to writing; AP: conceived and supervised research, collected field data, and wrote the paper. All authors read and approved the final manuscript.
